# Introduction of medication review and medication report in Swedish hospital and primary care, using a theory-based implementation strategy

**DOI:** 10.1186/s12913-020-05696-3

**Published:** 2020-09-14

**Authors:** Siw Carlfjord, Eva Malmberg, Carina Skoglund

**Affiliations:** 1 Department of Health, Medicine and Caring Sciences, Division of Community and Heath, Linköping University, SE-58183 Linköping, Sweden; 2 Department of Clinical Pharmacology, Region Östergötland, SE-581 91 Linköping, Sweden; 3 Centre for Healthcare Development, Region Östergötland, SE-581 91 Linköping, Sweden

**Keywords:** Quality improvement, Medication report, Medication review, Implementation

## Abstract

**Background:**

The development of routines regarding medication is important to avoid medication-related harm. Medication review and medication reports have earlier been found to be effective, but their implementation is not always successful. The aim of this study was to evaluate the introduction of medication review/medication report in hospital and primary care, in terms of perceptions of the implementation strategy, adoption and sustainability, in one Swedish county.

**Methods:**

The study included 105 clinics. Data was collected from interviews with managers immediately after implementation, survey data and registry data collected five years later. Quantitative data was analysed using non-parametric statistical tests. Open-ended questions were analysed with qualitative methods.

**Results:**

The implementation activities were found satisfying, and managers were satisfied with their own influence over the process. After five years medication review and medication reports were reported mainly implemented by the managers. Facilitating factors reported were routines, staff influence, dedication, reminders, and a stable workforce, while hindering factors reported were organizational factors, less commitment and flaws in reporting. Registry data showed that performance of medication review was very limited in primary care. In hospital care medication review was registered in about one fifth of the patients, while medication reports, only relevant for hospital care, was registered in half of the patients.

**Conclusions:**

The managers’ perceptions of the implementation process were mainly positive, and they found the new practices of medication review/medication report implemented. Implementation success, however, was not supported by registry data, showing the need for reliable outcome measures for implementation.

## Background

The implementation of new methods with the purpose of improving care, patient safety and patient outcomes is highly prioritized in health care organizations all over the world [1]. Nevertheless many innovative interventions or suggested improvements have difficulties in being spread, adopted and sustained in practice. One area, often mentioned to be in need of improvement, is the development of routines regarding medication [2, 3]. Medicines are the most commonly used therapies in health care, new drugs are continuously developed and the consumption is increasing [4]. Generally drug treatment has beneficial effects, but medication therapy may also be associated with negative health outcomes. If medicines are prescribed or used inappropriately, patient safety can be compromised, treatment will result ineffective and it can ultimately lead to medication-related problems [5, 6].

A method suggested in order to reduce medication-related harm is the systematic assessment of the individual patient’s pharmacotherapy, a process called medication review [7]. At admittance to hospital, or at a general practitioner consultation in primary care, the patient’s current medications are identified and compared to the registered list, unintended discrepancies are reconciled and the list is updated [8]. Medication reviews have been put into practice in many countries, however, the implementation into routine health care has been challenging [9]. At discharge from hospital a medication report is proposed to be handed over to the patient, in order to reduce patient-related mistakes regarding medication distribution at home [10, 11].

In Sweden the National Board of Health and Welfare introduced medication review, and also medication reports nationally in 2012, with the aim of reducing inappropriate prescribing and preventable medication-related problems [12]. Patients aged 75 years or older with at least five prescribed medications are entitled to receive medication review once a year in primary care, and when admitted to hospital. Medication reports should be provided at discharge from hospital. In Östergötland county the local health authorities decided to apply a broader scope in that medication review must be conducted for all patients irrespective of age or number of medications. Implementation efforts were put into practice in 2013 with the intention to establish “uniform procedures in order to reduce the risk of avoidable medication-related problems” [13]. The strategy used for the implementation was closely linked to the Quality Implementation Framework described by Meyers et al. [14], and is described in detail in the Methods section.

Proctor et al. suggest that when studies evaluating implementation are reported, the strategies used should be thoroughly labelled and described, and operational definitions should be provided [15]. In the present study we have considered these suggestions and try to report the implementation processes in sufficient detail, combining the Proctor et al. suggestions with the Quality Implementation Framework [14, 15].

The aim of this study was to evaluate the introduction of medication review/medication report in hospital and primary care in one Swedish county, in terms of perceptions of the implementation strategy, adoption and sustainability.

## Methods

This study applies a mainly quantitative design, including data from structured interviews immediately after the implementation activities, and survey data collected five years later. Cross-sectional registry data from one month during the fifth year after initial implementation were also included. The study is reported according to the Standards for QUality Improvement Reporting Excellence (SQUIRE) 2.0 checklist [16].

### Setting

The study was performed in the county of Östergötland, Sweden, and includes all the 105 clinics operating in the area, hospital and primary care, both private and public. In Sweden health care is mainly publicly funded, and the private clinics included operate with public funding according to local agreements with the authorities. The county of Östergötland has approximately 450,000 inhabitants and has been found to be representative for the whole country in terms of age distribution, employment rates and proportion of rural and urban areas. The health care authorities divide the county into Western, Eastern and Central Östergötland, with one hospital and a number of primary health care centres operating in each part.

### Implementation strategy

The strategy used for the implementation of medication reviews in Östergötland county was closely linked to the Quality Implementation Framework described by Meyers et al., consisting of four phases and 14 critical steps [14]. The implementation activities are described in Table 1, with references to the framework. During the implementation phase there were challenges in terms of lack of continuity in one of the three country parts, due to staff turnover.

**Table 1:** Implementation strategy based on the Quality Implementation Framework (Meyers et al. 2012)

Quality Implementation Framework (QIF)			Application in project	
**Phase**	**Step category**	**QIF step according to phase**	**Definition**	**Operationalization**
Phase One: Initial considerations regarding the host setting	Assessment strategies	1–3. Conducting a needs and resources assessment, a fit assessment, and a capacity/readiness assessment	Initial assessment	Initial assessments were not considered applicable, as the initiative for implementing Medication Review and Medication Report was a governmental decision based on scientific findings.
	Decisions about adaptation	4. Possibility for adaptation	Adaptation	All units were allowed to decide on local routines in addition to the general routine that was implemented.
	Capacity-building strategies	5. Obtaining explicit buy-in from critical stakeholders	Decision	A decision of systematic implementation of Medication Review and Medication Report in Health Care in the county council of Östergötland, was made at the county council management level.
		6. Building general/organizational capacity	Steering group	A steering group including the head health care manager, medical directors and health care managers from different sectors was recruited to supervise the implementation.
		7. Staff recruitment/maintenance	Staff	Recruitment of a project manager, and delegates representing the three parts of the county, the department of clinical pharmacy, and medical doctors from primary care and hospital care. An implementation researcher was also invited to the group.
		8. Effective pre-innovation staff training	Guideline available	Guidelines for Medication Review and Medication Report were developed and made available for staff at the internal website.
Phase Two: Creating a structure for implementation	Structural features for implementation	9. Creating implementation teams	Teams	One implementation agent (IA) in each part of the county was assigned to lead the activities.
		10. Developing an implementation plan	Implementation plan / Communication plan	A local implementation plan was developed, including the following steps:IA contacts the manager, local timetable is set IA meets all physicians at the unit, a “physician in charge” is assignedSecond physician meeting, including information about documentation The manager is in charge of informing the nurse group.
Phase Three: Ongoing structure once implementation begins	Ongoing implementation support strategies	11. Technical assistance/coaching/supervision	Electronic medical record system	The medical record system was developed in order to facilitate performance and reporting of Medication Reviews and Medication Reports.
		12. Process evaluation	Post-implementation interview	Structured interviews with managers when all the facilitating activities had been completed
		13. Supportive feedback mechanism	Feed-back	Follow-up data on unit level was made available to all managers, intended to increase adoption and sustainability
Phase Four: Improving future applications		14. Learning from experience	Follow-up survey	At follow-up after five years, facilitating and impeding factors were identified, based on open-ended questions

### Data collection

Data were collected when the implementation activities were completed, and five years later. Registry data based on the reporting of medication reviews and medication reports were collected from one month during the fifth year.

#### Interviews

Structured interviews following an interview guide developed for the project (Additional file 1), were performed with the department managers as the last step of the implementation activity. A meeting was arranged with the manager, the questions were put orally, and a protocol was completed by the interviewer. The questions concerned perceptions of the implementation activities, the support from the implementation team, if practice had changed and the managers view of how medication reviews and medication reports were perceived by physicians and other staff members. Questions were mainly multiple choice, but with an option to make comments.

#### Survey

Five years after the first interview, in spring 2019, the department managers were approached by e-mail and asked to fill out a survey regarding the implementation of medication reviews/medication reports at their department. The survey was distributed to the current manager, meaning that it could be another person than the one who was interviewed in the first place. Survey questions were developed for the specific study, based on the former interview questions, and were discussed with county council representatives and patient safety experts to obtain face validity. The survey also included open ended questions regarding facilitating factors where medication review/medication reports had been successfully implemented, and perceived hindering factors where it was partly or not at all implemented. The questionnaire can be found in Additional file 2.

#### Registry data

Data on performance are automatically transferred from medical records, and stored in the county council database. Data were available for a majority of the participating clinics, and were used to quantitatively evaluate how medication review and medication report is actually performed at the clinics. As data showed very little fluctuation over time, cross-sectional data from one month in year five, coinciding with the follow-up survey, were used for the analysis.

### Data analysis

Quantitative data from the structured interviews and the survey, as well as registry data. Were analysed using the Statistical Package for the Social Sciences (SPSS) version 24. Data from the different county parts were compared using the Mann Whitney U test, and changes over time were analysed using the Chi-Square test. Correlations were calculated using Spearman’s rho.

Data from the open ended questions in the survey were analysed according to the method Qualitative Content Analysis with a deductive approach, so called Directed Content Analysis [17]. The statements were categorized according to the four domains Context, Adopters, Implementation object and Implementation strategy, often used as determinants for successful implementation in the description and analysis of implementation processes [18, 19].

## Results

### Response rates

For the follow-up interview 105 managers were contacted, and 90 (86%) of these agreed to participate in the interview. The survey distributed five years later was sent to the same 105 clinics, and yielded answers from 54 clinics (51%), (32 hospital care, 22 primary care). Registry data was available from 61 of the participating clinics (29 hospital care, 32 primary care). Data from all data sources was available for 31 clinics (21 hospital care, 10 primary care).

### Results from follow-up interviews

#### Managers perceptions of the implementation strategy and activities

Table 2 shows the managers’ opinions about the implementation. Overall satisfaction with the implementation was reported by most of the participating managers, and a majority reported that the support from the implementation team had been good. There was, however a difference between the three county parts, A, B and C, with managers in A less satisfied than the others. No or small influence over the implementation process was reported by 80%, but 81% of the responding managers were satisfied with the influence they had. Less satisfaction with the influence was found in county part A. County part A was the one that had problems with staff turnover during the implementation process.

**Table 2:** Manager opinions about implementation, implementation support and influence

	Total n (%)	County part A n (%)	County part B n (%)	County part C n (%)	Difference between county parts*
Are you satisfied with how medication review/medication report was implemented at your clinic/centre?					*p* > 0.05
Totally satisfied	34 (39)	7 (24)	20 (44)	7 (50)	
Quite satisfied	44 (50)	19 (66)	18 (40)	7 (50)	
Dissatisfied	10 (11)	3 (10)	7 (16)	0	
Very dissatisfied	0	0	0	0	
How did you perceive the support from the implementation team?					C > A (*p* < 0.05) B > A (*p* < 0.01)
Good	62 (72)	12 (44)	38 (84)	12 (86)	
Quite good	13 (15)	7 (26)	4 (9)	2 (14)	
Quite bad	5 (6)	3 (11)	2 (4)	0	
Bad	6 (7)	5 (18)	1 (2)	0	
Did you perceive having an opportunity to influence the implementation process?					p > 0.05
Totally	5 (6)	1 (4)	2 (4)	2 (14)	
Quite much	13 (15)	4 (14)	5 (11)	4 (27)	
Somehow	35 (40)	8 (29)	23 (50)	4 (27)	
Not at all	35 (40)	15 (54)	16 (35)	4 (27)	
Are you satisfied with the influence you had over the implementation process?					C > A (p < 0.05) B > A (p < 0.05)
Totally satisfied	25 (35)	4 (17)	16 (43)	5 (50)	
Quite satisfied	33 (46)	12 (50)	16 (43)	5 (50)	
Dissatisfied Very dissatisfied	9 (13)	5 (21)	4 (11)	0	
	4 (6)	3 (12)	1 (3)	0	

* Calculated using the Mann-Whitney U test

#### Managers perceptions of staff attitudes regarding medication review/report

Among the managers, 53% reported that the physicians were positive to medication review/report, and 48% found other staff groups positive.

#### Change in practice

A change in practice was reported by 61% of the managers, 33% reported no change, and 6% did not answer the question.

### Sustainability according to survey data

After five years, 46% of the responding managers considered medication review implemented at their clinic, and another 48% reported it to be partly implemented. Medication reports, relevant only for hospital clinics, was considered implemented at 42% of these clinics, and partly implemented at another 35% of the clinics. No differences could be found according to part of the county.

After five years the attitudes among physicians and staff were reported slightly, but not significantly, more positive than at the time for the follow-up interview, as displayed in Table 3.

**Table 3:** Attitudes among physicians and other staff members at follow-up and after five years, as reported by managers

	Attitudes at follow-up n (%)	Attitudes after 5 years n (%)	Change in attitude over time**
How would you describe the opinion regarding medication review and medication report among the physicians at your clinic/centre?*			5 years > follow-up (p > 0.05)
Very positive	1 (2)	2 (5)	
Positive	20 (48)	23 (55)	
Neither positive nor negative	14 (33)	15 (36)	
Negative	5 (12)	2 (5)	
Very negative	2 (5)	0	
How would you describe the opinion regarding medication review and medication report among other staff members at your clinic/centre?*			5 years > follow-up (p > 0.05)
Very positive	3 (8)	3 (8)	
Positive	15 (39)	24 (63)	
Neither positive nor negative	19 (50)	11 (29)	
Negative	1 (3)	0	
Very negative	0	0	

*Only clinics/centres where data was available from both data collections

**Calculated using the Pearson Chi-square test

### Facilitating and hindering factors

Facilitating or hindering factors were described by the managers in the five year survey. The results are presented according to the categories Context, Adopters, Implementation object and Implementation strategy.

#### Factors perceived to have facilitated implementation of medication review/report

Regarding context, structural factors such as a stable workforce and a reasonable work load, were mentioned as facilitating factors. For medication review the presence of a pharmacist was also considered important. Where routines were partly in place before the formal implementation was initiated, and performing medication review was considered part of the task, this was described as facilitating.

Where implementation was described as successful, staff members (the adopters), had taken part in discussions and decisions. According to the managers, staff were dedicated, had a positive attitude to performing medication review/report and found the task important. The task was also perceived as being easy to fulfil.

Regarding the strategy, the development of routines and continuous reminders was considered crucial, together with information and communication with staff. The implementation activities were found to be well balanced, and at some clinics ongoing improvement projects were also mentioned as facilitating factors.

#### Factors perceived to have hindered implementation

At clinics where medication review or medication report was described as partly implemented the hindering contextual factors mentioned were staff shortage, lack of time and how work was organized. Frequent changes in practice, initiated from management level, was also mentioned.

A lack of commitment regarding medication review/report among staff members (adopters) was mentioned, but also flaws in reporting, which means that more activities were believed to be performed than what is actually reported. Regarding medication review a lack of competence among staff was also mentioned. A strategy factor mentioned was the need for continuous reminders and feed-back, which sometimes were not in place.

At some clinics medication review or medication reports was not considered relevant, as the patients are young, not severely ill, and have very few prescribed medicines. Some clinics only serve outpatients, making medication reports irrelevant.

### Registry data

Registry data was available for 32 primary care clinics, and the proportion of patients who had had a medication review ranged from 0.5–12% (median8%). Data on medication review from hospital care was available from 29 clinics, and the proportion of patients who had had a medication review ranged from 1 to 97% (median 32%). Data on medication reports was available from 26 of the hospital clinics. The proportion of patients who had received a medication report at these clinics ranged from 3 to 88% (median 58%). Data regarding medication report is not relevant for primary care.

### Comparison of data from different sources

#### Primary care

Data from all the three data sources was available from 10 primary care clinics, and are displayed in Table 4. The managers in primary care were positive or very positive to medication review, they were mainly satisfied with the implementation efforts, and with the support. Six of the managers reported that medication review was successfully implemented. However, according to registry data, no clinic had a proportion higher than 8% of patients having a medication review. Managers’ report on implementation did not correlate to proportion of patients having a medication review according to the register (r = 0.11. *p* = 0.8).

**Table 4:** Medication review in primary care, sorted according to proportion of patients with medication review

Unit*	Satisfied with implementation^**1**^	Opinion about support^**1**^	Medication review implemented^**2**^	Opinion about medication report/review^**2**^	Proportion with medication review (%)^**3**^
P1	Quite satisfied	Good	Partly	Very positive	1
P2	Quite satisfied	Quite bad	Yes	Positive	1
P3	Totally satisfied	Good	Partly	Very positive	1
P4	Quite satisfied	Quite good	Yes	Positive	2
P5	Totally satisfied	Good	Yes	Positive	3
P6	Not satisfied	Good	Yes	Positive	4
P7	Totally satisfied	Good	Partly	Very positive	4
P8	Quite satisfied	Good	Yes	Positive	7
P9	Totally satisfied	Good	Partly	Very positive	7
P10	Totally satisfied	Good	Yes	Very positive	8

^1^Data from follow-up interview^2^Data from 5 year survey^3^Data from county register

*P1-P10 = Primary Care Unit 1–10

#### Hospital care

Data regarding medication review from all the three data sources was available from 20 hospital care clinics, and are displayed in Table 5. The vast majority of the managers stated that they were satisfied with the implementation efforts and the support provided, and 17 of the 20 managers stated that they were positive or very positive to medication review/report. They also stated that medication review was implemented (13) or partly implemented (6). The correlation between the managers´ report of implementation and the proportion of patients registered to have had a medication review was, however, very weak and not significant (r = 0.13, *p* = 0.6). Nor did the managers opinion about medication review/report and registry data correlate.

**Table 5:** Medication review and medication report in hospital care, sorted according to proportion of patients with medication review

Unit*	Satisfied with imple-mentation^**1**^	Opinion about support^**1**^	Medication review implemented^2**^	Opinion about medication report/review^**2**^	Proportion with medication review (%)^**3**^	Medication report implemented^2**^	Proportion with medication report (%)^**3**^
H1	Totally satisfied	Good	Partly	Very positive	3		
H2	Quite satisfied	Good	No	Neither positive nor negative	8	No	28
H3	Totally satisfied	–	Partly	Very positive	9	Partly	6
H4	Totally satisfied	Good	Yes	Very positive	9	Yes	18
H5	Quite satisfied	Bad	Yes	Positive	12	Yes	39
H6	Totally satisfied	Quite good	Yes	Positive	13		
H7	Totally satisfied	Good	Yes	Very positive	25	Yes	69
H8	Quite satisfied	Quite good	Yes	Positive	28	Yes	64
H9	Not satisfied	Good	Yes	Very positive	29	Yes	76
H10	Quite satisfied	Quite good	Yes	Very positive	32		
H11	Quite satisfied	Quite good	Partly	Positive	37		
H12	Quite satisfied	Good	Partly	Neither positive nor negative	38		
H13	Totally satisfied	Good	Yes	Neither positive nor negative	45	Yes	14
H14	Totally satisfied	Quite good	Yes	Very positive	46	Yes	73
H15	Totally satisfied	Good	Yes	Positive	58	Partly	58
H16	Quite satisfied	Good	Yes	Very positive	72	Yes	69
H17	Totally satisfied	Good	Partly	Positive	76	Partly	4
H18	Quite satisfied	Good	Yes	Positive	91	Yes	76
H19	Quite satisfied	Bra	Yes	Positive	94	Yes	13
H20	Quite satisfied	–	Partly	Very positive	97	Yes	77
H21	Quite satsfied	Good	Partly	Neither positive nor negative	–	No	75

^1^Data from follow-up interview^2^Data from 5 year survey^3^Data from county register*H1-H21 = Hospital clinic 1–21

**Medication review: Data from 20 units, Medication report: Data from 16 units

Regarding medication report, data from all three data sources was available from 16 clinics displayed in Table 5. Most of the managers stated that medication review was implemented (11) or partly implemented (3).

## Discussion

This study showed that the implementation activities provided when medication review and medication report was introduced were found satisfying by the managers, who also reported satisfaction with their own influence over the process. Five years after the initial implementation, medication review and medication reports were reported implemented by the managers in almost half of the clinics where relevant. Facilitating factors reported were routines, staff influence, dedication, reminders, and a stable workforce, while hindering factors reported were organizational factors, less commitment, flaws in reporting, and the absence of reminders or feed-back.

From the registers, however, the medical records show that performance of medication review was very limited in primary care. In hospital care medication review was registered in about one fifth of the patients, while medication reports, only relevant for hospital care, was registered in half of the patients.

The application of implementation theory in the planning and performance of the implementation activities in this study worked out well. The managers were satisfied with how the process had been facilitated by the implementation team, and, even though their influence over the activities was limited, they reported satisfaction also regarding influence. Former studies have shown that the implementation activities in terms of strategy and facilitation is important for implementation success [20, 21]. Despite this, few implementation studies clearly report in what way their strategies are informed by theory, and do not describe the strategies used [15].

Based on the manager reporting of fully or partly implemented medication review/medication report the implementation must be considered successful. If the practise was not applied at all before implementation, and now half of the clinics report it implemented this is a success. Based on registry data implementation outcome is not that successful, with low proportions receiving medication review, but higher proportions receiving medication report where relevant. From former studies, however, we know that implementation activities seldom result in full and sustainable change. Systematic reviews evaluating different implementation strategies show small to moderate effects, with effect sizes usually 5–12% [22–24].

Barriers and facilitators for implementation have been reported in a number of studies. However, barriers and facilitators are often context-specific, and cannot easily be transferred from one context to another. In the present study a facilitator mentioned was dedication. This could also be seen in terms of motivation [25]. Intrinsic motivation, meaning that the staff member has gained an understanding about the importance of practising the new working method, can lead to a dedication for the task. At clinics where this dedication is present the implementation has been more successful than where staff attitudes are described as lack of commitment to the task.

Another facilitator mentioned was reminders, which was not part of the overarching strategy, but was used locally by the managers. Reminders have also been identified as important to achieve change in practice [26]. Staff tend to forget tasks that are added to their routines, and the practice of reminders is a way to overcome this.

A hindering factor, or rather an explanation for low reported numbers, that was mentioned by the managers was flaws in reporting. This can also be an explanation for the great discrepancy between manager reporting of medication review/medication report being implemented at their clinic, and the very low proportions of patients receiving this service according to registers. Self-reporting is known to be a measure that can be affected by social desirability [27], if the reporter knows what is expected, and reports a little more generously than what is actually true. However, if reporting to the register is not carefully performed, also the registry data will suffer from considerable limitations.

The fact that neither self-reported data, nor registry data can be totally reliable accentuates the problem with outcome measures in implementation research. A number of outcome measures have been proposed, for example by Proctor et al. suggesting Acceptability, Adoption, Appropriateness, Costs, Feasibility, Fidelity, Penetration and Sustainability to be evaluated [28]. Most of these measures, however, are depending on either self-reporting to the researchers or data registered by staff members in medical records.

The need for better ways to measure and evaluate implementation has been recognized by implementation researchers over time, and instruments for implementation research are continuously being developed and tested [29]. Still, the use of standardised and reliable instruments is not always feasible in the specific setting where implementation takes place.

### Methodological considerations

This study has limitations that should be considered when interpreting the results. It was performed in one specific county in Sweden, where the implementation was initiated by the authorities, which may not be a situation that represents the whole country. The data collection where managers were interviewed face-to-face may have affected their answers, knowing that the interviewer was part of the implementation team. Self-reporting, as already discussed is also a method that may imply a limitation.

## Conclusions

The implementation process was perceived as positive by the managers, who also find the new practices of medication review/medication report implemented in their clinics to a quite high extent. Implementation success, however, is not supported by registry data, showing the need for the development of reliable outcome measures for implementation.

## Supplementary information

**Additional file 1.**

**Additional file 2.**

## Data Availability

The datasets generated and analysed during the current study are available from the corresponding author on reasonable request.

## Abbreviations

QIF: Quality Implementation Framework
P1-P10: Primary Care Unit 1–10
H1-H21: Hospital clinic 1–21
