# Supplementary material for: Introduction of medication review and medication report in Swedish hospital and primary care, using a theory-based implementation strategy
Source: BMC Health Serv Res. 2020 Sep 14;20:867. doi: 10.1186/s12913-020-05696-3 (PMC7489027; doi:10.1186/s12913-020-05696-3)
Supplement: Supplementary file 1 — Additional file 1. [file 12913_2020_5696_MOESM1_ESM.pdf]

Developed by: Siw Carlfjord
Approved by: Carina Skoglund

Structured follow-up interview

Developed by: Siw Carlfjord
Approved by: Carina Skoglund

Instruction:
The interviewer keeps the form in his/her hand, asks the questions and marks the respondent's answer. Comments and answers to open-ended questions are written in short at the form.

Are you satisfied with how medication review/medication report was implementated at your clinic/centre?

Totally satisfied	Quite satisfied	Dissatisfied	Very dissatisfied

Comments:…….

Are you satisfied with the implementation outcome (according to registers)?

Totally satisfied	Quite satisfied	Dissatisfied	Very dissatisfied

Comments: …….

How did you perceive the support from the implementation team?

Good	Quite good	Quite bad	Bad

Comments: …….

Did you perceive having an opportunity to influence the implementation process?

Totally	Quite much	Somehow	Not at all

Comments: …….

Are you satisfied with the influence you had over the implementation process? (to deepen the previous question)

Totally satisfied	Quite satisfied	Not satisfied	Not at all satisfied

Comments: …….

Developed by: Siw Carlfjord
Approved by: Carina Skoglund

Enablers and barriers to the implementation can be expected. Did you experience enablers and barriers as expected?

Totally Quite much Somehow Not at all

Comments: …….

How would you describe the opinion regarding medication review and medication report among the physicians at your clinic/centre?

Very positive Positive Neither positive nor negative Negative Very negative

Comments: ………

How would you describe the opinion regarding medication review and medication report among other staff members at your clinic/centre?

Very positive Positive Neither positive nor negative Negative Very negative

Comments: …….

Did practice at your clinic/centre change after implementation of medication review and medication report?

No, we practiced medication review and medication report before the project Yes, practice changed

Comments: …….

What is your overall opinion about the implementation process?

Worked well Should have been performed in a different way

Example: More meetings, more education, other competencies in implementation team…

Comments: …….

Any other comments?
