# Supplementary material for: Introduction of medication review and medication report in Swedish hospital and primary care, using a theory-based implementation strategy
Source: BMC Health Serv Res. 2020 Sep 14;20:867. doi: 10.1186/s12913-020-05696-3 (PMC7489027; doi:10.1186/s12913-020-05696-3)
Supplement: Supplementary file 2 — Additional file 2. [file 12913_2020_5696_MOESM2_ESM.pdf]

Medication and patient safety

When the project ”Rätt Läkemedel” was completed five years ago, managers at hospital clinics and primary health care centres were interviewed regarding how they perceived the implementation of medication review and medication report. The aim of this study is to evaluate how these practices are applied today, and if the implementation can be considered successful. This is a follow-up study, and collected data will be used for internal purposes, but also in research on implementation/patient safety in collaboration with Linköping University. Any published research data will be presented so that no individual can be identified.

Background questions

In which part of the county is your clinic/centre located?

- ☐ Western part
- ☐ Central part
- ☐ Eastern part

Please state which clinic/centre you are representing.

Questions about practice

Do you consider medication review implemented at your clinic/centre?

- ☐ Yes
- ☐ Partly
- ☐ No
- ☐ Not applicable

What factors have facilitated the implementation?

What factors have hindered the implementation?

If you stated ”not applicable”, please tell us why you do not find it applicable.

Do you consider medication report implemented at your clinic/centre?

- [ ] Yes
- [ ] Partly
- [ ] No
- [ ] Not applicable

What factors have facilitated the implementation?

What factors have hindered the implementation?

If you stated ”not applicable”, please tell us why you do not find it applicable.

Do you apply the Region Östergötland guidelines for medication report and medication review?

- [ ] Yes
- [ ] Partly
- [ ] No

Is there an additional local routine for medication review and medication report at your clinic/centre?

- [ ] Yes
- [ ] No

How would you describe the opinion regarding medication review and medication report among the physicians at your clinic/centre?

- ☐ Very positive
- ☐ Positive
- ☐ Neither positive nor negative
- ☐ Negative
- ☐ Very negative
- ☐ Cannot respond

How would you describe the opinion regarding medication review and medication report among other staff members at your clinic/centre?

- ☐ Very positive
- ☐ Positive
- ☐ Neither positive nor negative
- ☐ Negative
- ☐ Very negative
- ☐ Cannot respond

What is your personal opinion about applying medication review and medication report?

- ☐ Very positive
- ☐ Positive
- ☐ Neither positive nor negative
- ☐ Negative
- ☐ Very negative

Thank you for your participation!
